# Stingless Bee Honey Improves Spatial Memory in Mice, Probably Associated with Brain-Derived Neurotrophic Factor (BDNF) and Inositol 1,4,5-Triphosphate Receptor Type 1 (*Itpr1*) Genes

**DOI:** 10.1155/2019/8258307

**Published:** 2019-12-02

**Authors:** Mohd Zulkifli Mustafa, Fairuz Nabila Zulkifli, Ivanna Fernandez, Abdul Razak Mariatulqabtiah, Muthuraju Sangu, Johari Nor Azfa, Mahaneem Mohamed, Nurhidayah Roslan

**Affiliations:** ^1^Department of Neuroscience, School of Medical Sciences, Universiti Sains Malaysia (USM), 16150 Kubang Kerian, Kota Bharu, Kelantan, Malaysia; ^2^Department of Cell and Molecular Biology, Faculty of Biotechnology and Biomolecular Sciences, Universiti Putra Malaysia (UPM), 43400 Serdang, Selangor, Malaysia; ^3^Laboratory of Halal Science Research, Halal Products Research Institute, Universiti Putra Malaysia (UPM), 43400 Serdang, Selangor, Malaysia; ^4^Malaysia Genome Institute (MGI), Jalan Bangi, 43000 Kajang, Selangor, Malaysia; ^5^Department of Physiology, School of Medical Sciences, Universiti Sains Malaysia (USM), 16150 Kubang Kerian, Kota Bharu, Kelantan, Malaysia

## Abstract

This study was conducted to evaluate the effects of stingless bee honey (SBH) supplementation on memory and learning in mice. Despite many studies that show the benefits of honey on memory, reports on the nootropic effects of SBH are still lacking, and their underlying mechanism is still unclear. SBH is a honey produced by the bees in the tribe of Meliponini that exist in tropical countries. It features unique storage of honey collected in cerumen pots made of propolis. This SBH may offer a better prospect for therapeutic performance as the previous report identifies the presence of antioxidants that were greater than other honey produced by *Apis* sp. In this study, SBH was tested on Swiss albino mice following acute (7 days) and semichronic (35 days) supplementation. Experiments were then conducted using Morris water maze (MWM) behaviour analysis, RT-PCR for gene expression of mice striatum, and NMR for metabolomics analysis of the honey. Results indicate spatial working memory and spatial reference memory of mice were significantly improved in the honey-treated group compared with the control group. Improved memory consolidations were also observed in prolonged supplementation. Gene expression analyses of acutely treated mice demonstrated significant upregulation of BDNF and *Itpr*1 genes that involve in synaptic function. NMR analysis also identified phenylalanine, an essential precursor for tyrosine that plays a role at the BDNF receptor. In conclusion, SBH supplementation for seven days at 2000 mg/kg, which is equivalent to a human dose of 162 mg/kg, showed strong capabilities to improve spatial working memory. And prolonged intake up to 35 days increased spatial reference memory in the mice model. The phenylalanine in SBH may have triggered the upregulation of BDNF genes in honey-treated mice and improved their spatial memory performance.

## 1. Introduction

Stingless bee honey, also known as meliponini, trigona, sugar bag, or pot honey, is a precious product of the stingless bee, which also produces propolis and fermented pollen. The honey is produced by the stingless bees (Hymenoptera, Apidae, and Meliponini), which are the largest groups of eusocial bees on earth and can be found in most tropical and subtropical regions of the world such as Brazil and other parts of South America, Africa, Southeast Asia, and Northern Australia [1]. The bee is not commonly fed and flies within a range of 1000 meter radius to extract floral nectars and nonfloral nectars and sap from flowers, leaves, tree trunks, and fresh fruits. The production of this honey is known for its distinct flavour and aroma, with a fluid texture and slow crystallization [2]. Despite producing multifloral honey, the honey production has ensured a reproducible honey quality because the bees are locally adapted to surrounding, and foraging is done in a short range.

**Figure 1:**
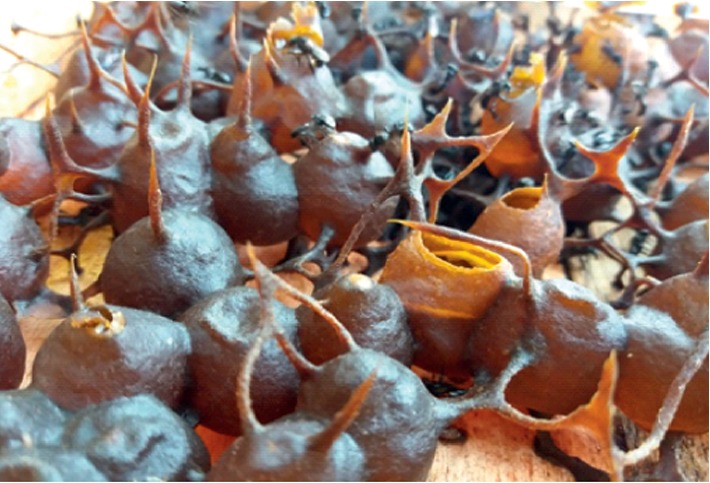
Propolis cerumen pots built by stingless bees (*H. itama*) to keep their honey.

The stingless bee offers unique features of honey storage. Different from *Apis* sp. bees that store honey in hexagonal-shaped honeycombs, stingless bees store honey in clusters of small propolis cerumen pots, as shown in Figure 1. In Malaysia, the stingless bee is known as *Kelulut*, and *Heterotrigona itama* (*H. itama*) is the main species reared due to the easy domestication process as well as the size of the pots that enable absolute honey suction as compared with other species that need pots squeezed upon harvesting. Locally, stingless bee honey production surpasses the *Apis* sp. honey to provide a newly developed and alternative honey source. The physicochemical and antioxidant properties reported from this honey also include the presence of flavonoids and carotenoids, and it is higher than other various types of Malaysian honey produced by the *Apis* sp. variety more commonly known in honey production [3].

A growing interest in the study of the physicochemical composition of stingless bee honey has led to various studies and research on the potential medicinal properties of this honey. In the past decade and until now, research has been actively done to investigate the medicinal potential of this stingless bee honey. Among established reports are evidence of its anticancer, anti-inflammatory, wound healing and antimicrobial properties, antioxidant activity, use in cataracts studies, and effects on antiobesity [1, 4–9]. However, despite positive studies reported on honey bees from *Apis* sp., there is still a lack of investigation on the neurological effects of stingless bee honey. Hence, this gap requires more research and investigation.

Several studies of honey from *Apis* sp. suggest that honey provides neuroprotective and nootropic effects. Honey possesses a memory-enhancing effect [10] and anxiolytic, antinociceptive [11, 12], anticonvulsant, and antidepressant effects [12] and has potential to improve the oxidative status of the brain [13, 14]. The nootropic effects of honey supplementation were observed in the development of the brain in infants and demonstrated improvements in memory and intellectual performance of children [15]. A recent study also reported that the honey from *Apis dorsata* enhanced hippocampal pyramidal count and spatial memory performance of adult male rats [16]; hence, research results show that honey can potentially act as a natural brain supplement to prevent neurodegeneration.

The molecular signalling presentation in memory is extensively studied in the adult central nervous system (CNS) via the neurotrophin pathway. The neurotrophin signalling through brain-derived neurotrophic factor (BDNF) and TrkB receptors regulate proliferation, cell survival, dendrite and axon growth, and the fate of neural precursors [17, 18]. The interaction of BDNF and TrkB is important for synaptic activity regulation and plasticity [19]. Both are thought to be responsible in hippocampal learning and memory formation. It is also vital in influencing somatic growth, dendritic complexity, and spine density in striatal neurons [20].

BDNF is crucial for long-term brain plasticity through regulation of NMDAR phosphorylation by enhancing glutamatergic synaptic transmission at the postsynapse [21]. BDNF release is supported by the BDNF-induced increase in NMDAR subunit, creating more opportunity for Ca^2+^ influx. Controls by Inositol 1,4,5-triphosphate receptor (*Itpr*) genes, IP_3_ activity, and intracellular Ca^2+^ release are boosted when there is a rise in TrkB signalling, thus contributing to the enhancement of the cycle [18]. The receptor for the IP_3_ pathway, which is encoded by the *Itpr*1 gene, became an intracellular IP_3_-gated calcium channel that modulates intracellular calcium signalling, hence influencing the survival of sympathetic neurons [22]. A study identified the consistent upregulation of *Itpr*1 gene lasting for few days in working memory tasks [23]. Hence, the increase of BDNF and *Itpr* expression in long-term potentiation is thought to underlie the process of learning and memory [21].

Therefore, this study was conducted to evaluate the potential of stingless bee honey to improve learning and memory in mice, specifically via MWM behaviour analysis as well as the BDNF and *Itpr*1 gene expression study. Collectively, the data in this study provides preliminary evidence suggesting that stingless bee honey treatment provides a positive role in improving memory performances in female mice.

## 2. Materials and Methods

### 2.1. Honey Samples

A freshly harvested stingless bee (*H. itama* sp.) honey sample was collected. The sample was collected directly from a commercial MUSTAFA-hive into a sampling bottle. The hive is designed to effectively promote more hygienic honey production [24]. The collected sample was protected from direct sunlight and kept at 4°C. The sample was brought to room temperature before dilution for supplementation to mice.

### 2.2. Chemicals and Reagents

Paraformaldehyde (PFA) and chloroform were obtained from Merck Milipore, USA. NaHPO_4_, NaH_2_PO_4_, and isopropyl alcohol were obtained from Sigma-Aldrich, Germany. TRIzol reagent (Invitrogen), SuperScript™ IV First-Strand Synthesis System, SuperScript™ IV Supermix, 1 kB Plus DNA Ladder, 10X Blue Juice™ Gel Loading Buffer, Agarose powder, and 10X TBE Buffer were purchased from Thermo Fisher Scientific, USA. DORMINAL 20% (200 mg Pentobarbital Sodium) (Alfasan, Holland), Ethanol denatured (HmbG, Germany), NMR buffer (KH_2_PO_4_ and sodium azide), and Trimethylsilylpropanoic acid (TSP) were used as a reference signal for NMR spectra. All chemicals and reagents used were of analytical grade.

### 2.3. Animals

Female Swiss albino mice (*n*=35) of age 2.5 months and weighing approximately 25 to 30 g were obtained from Animal Research and Service Centre (ARASC), Universiti Sains Malaysia (USM), Malaysia. The mice were randomly divided into five groups with seven mice per group and were kept in one standard polypropylene cage. All mice were housed in the animal centre, under 12 h light-dark cycles, and maintained at room temperature. Food and water were accessed freely and provided *ad libitum*. Cages and bedding were changed routinely to ensure a clean environment. All mice were acclimatized for one week prior to treatment and testing. The experimental protocol was approved by the Animal Ethics Committee of USM (No. of Animal Ethics Approval: USM/Animal Ethics Approval/2015/(97) (691)).

### 2.4. Honey Treatment Dose and Mice Grouping

Animals (*n*=35) were divided into one control and four treatment groups (*n*=7 in each group). Treatment groups were subdivided into two acute treatment (seven days) and two semichronic (35 days) groups. Each acute treatment and semichronic group received a low dose (750 mg/kg) and high dose (2000 mg/kg) of honey supplementation.  Table 1 summarizes the experimental groups used for this study. The dose selection was made according to a 162 mg/kg of honey dose for human [25] converted into a mice dose using a formula [26] as mentioned below:

Human equivalence dose (HED), mg/kg = Animal dose, mg/kg × Correction factor (*K*_m_) ratio.

*K*_m_ ratio for mice = 0.081.

During treatment, 0.3 ml of honey dilution was force-fed daily per mouse via oral gavage. For the control group, each mouse was also gavaged with 0.3 ml of ultrapure water daily during the treatment period. All mice were labelled and weighed before treatment.

### 2.5. Morris Water Maze (MWM)

**Figure 2:**
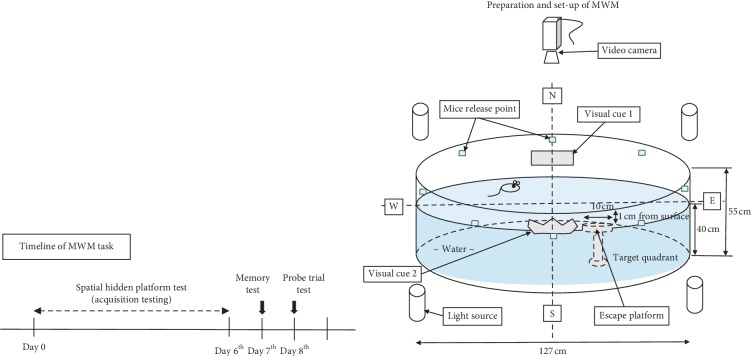
Timeline for the Morris water maze (MWM) task and preparation and set-up for MWM test.

The MWM test was done according to methods from previously reported studies [27–29]. The test consists of preparation and habituation to MWM, followed by a learning and memory behavioural task. This task was done after the completion of acute and semichronic treatment. It consists of a six day trial to learn the hidden platform's position, a memory test on the 7^th^ day, and a probe trial test on the 8^th^ day. This test was conducted in a quiet behaviour room.  Figure 2 shows the timeline for the task and preparation and set-up for this test. The apparatus used in the MWM consisted of a black circular tank with a diameter of 127 cm and a depth of 55 cm. The tank was filled to a depth of 40 cm with water, and the temperature was kept between 25 to 27°C. The tank was divided into four quadrants of the same area, which are Northeast (NE), Northwest (NW), Southeast (SE), and Southwest (SW). An escape platform about 10 cm in diameter was submerged 1 cm below the water surface in one of the quadrants (target quadrant). Several distal cues were provided around the tank to guide the mice as it navigated within the pool. A video camera was mounted directly above the water maze to record the activity of the mice during testing.

Before testing with MWM, all mice were habituated in water where they were allowed to swim in the tank without a platform for 60 seconds. After habituation, a spatial hidden platform test (acquisition testing) was done for six days during the learning phase and a memory test on the seventh day. In this test, each mouse received three trials per day for seven consecutive days. The starting position (N, E, S, and W) was randomly changed in every trial. During each trial, each mouse was released in the water with its head pointed toward the sidewall and was given 60 s to find the hidden platform. If the mouse found the platform, it was allowed to remain on it for 15 s. If the mouse was unable to locate the platform within 60 s, it was guided to the platform and also allowed to stay on it for 20 s. During this acquisition phase, the platform position remained unchanged (SE quadrant). The spatial memory and learning of this experiment rely on the time taken by the mice to find the platform. Hence, the activity of each mouse was recorded, and their escape latency was calculated and analysed. On the 8^th^ day of the experiment, a probe trial test was done where the platform was removed, and all mice were released from the same starting position, north (N). Activity of the mouse was then recorded for 60 s. The frequency of visit and time spent in the quadrant of the previous platform (SE quadrant) within those 60 s were recorded and analysed for spatial reference memory.

### 2.6. Brain Sample Collection and Tissue Homogenate Preparation

After completion of the behaviour study, the brain of all control and honey-treated mice was then collected. Under anaesthesia, the mice were perfused via cardiac puncture with 4% PFA in PBS at pH 7.2. The head was then decapitated, and the brain was taken out. For the gene expression study, the striatum was identified and then dissected out according to the method described by Haron et al. [30]. The harvested striatal region was homogenized in a TRIzol reagent (Invitrogen). RNA was extracted and precipitated with chloroform and phenol before stored at −80°C for further analysis.

### 2.7. Gene Expression Analysis

In sample preparation, the TRIzol reagent (Invitrogen) was added and the striatum were tattered, and then RNA was extracted and precipitated with chloroform and phenol and stored at −80°C until used. RNA quality was then assessed by bioanalysis (2100, Agilent Technologies, Santa Clara, CA) [31]. The current study was carried out with modifications from Fedorenko et al. [32] using specific primers with the following sequences as listed in Table 2.

The primers were checked for uniqueness in BLAST software online and were selected for the fragment of gene BDNF and *Itpr1* of the mouse (number of gene in the NCBI database). Each sample was analysed three times. The length of the product was checked using electrophoresis in 1% agarose gel. Changes in the level of expressions of BDNF, *Itpr1*, and the level of expression of gene *Actb* (gene of *ß*-actin, used as a housekeeping gene) were presented. The intensity of each gene was then calculated using ImageJ.

### 2.8. Metabolomics Analysis by Nuclear Magnetic Resonance (NMR)

In this experiment, the nutritional content of the stingless bee honey sample used for the supplementation of mice was further analysed using the ^1^H NMR spectroscopy method as described by Ramli et al. [33]. Prior to testing, the water content for the honey sample was first measured using a hand-held refractometer. In this test, the ^1^H NMR spectra were recorded at 300 K on a Bruker Ascend 700 MHz NMR spectrometer (Bruker Biospin, Germany) equipped with a 5 mm triple-resonance probe (700.4 MHz ^1^H frequency). A total of 40 scans and four prior dummy scans of 65*k* points were acquired with a spectral width of 15.985 ppm, a receiver gain of 8, and an acquisition time of 1.46 s. The standard one-dimensional NOESY-presaturation pulse sequence suppressed the water resonance. All NMR spectra were manually phased, baseline-corrected, and calibrated by the trimethylsilylpropanoic acid (TSP) signal at 0.0 ppm using topsin 3.0. The NMR spectral data analysis was then performed using targeted profiling with Chenomx NMR Suite 8.1 software for metabolomics analysis. This analysis compares the integral of a known reference signal (TSP) with signals derived from a library of compounds containing chemical shifts and peak multiplicities.

### 2.9. Statistical Analysis

The data for behavioural analyses were expressed as mean ± standard error of means (SEM) and were analysed using SPSS (Statistical Packages for Social Science 22.0) and MS Excel 2013. One-way analysis of variance (ANOVA) followed by Tukey's post hoc test was used to compare the escape latency in the spatial hidden platform test and frequency of visit and time spent in the previous quadrant in the probe trial test, between all honey-treated groups and control group mice. An independent *t*-test was also used to compare the escape latency between each honey-treated group with the control and also between two dosages of acute and semichronic honey-treated mice. All data were considered to be significantly different if the *p* value was less than 0.05 (*p* < 0.05). For the gene expression study, ANOVA was also conducted to find a significant difference between groups and was further tested with Tukey's post hoc test.

## 3. Results

### 3.1. Learning and Memory Behavioural Analysis

**Figure 3:**
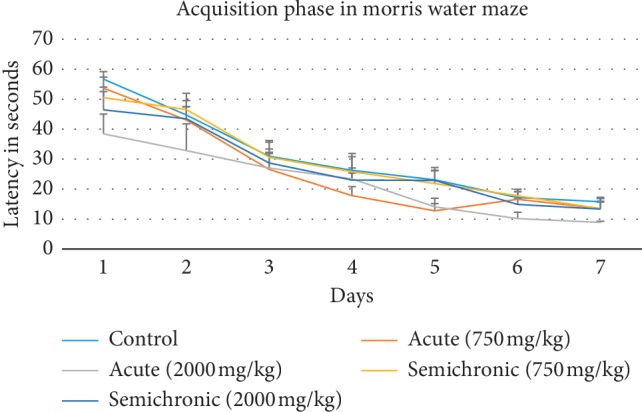
Escape latency for seven days of the acquisition phase in control and honey-treated groups using the spatial hidden platform test. Data are represented as mean ± SEM, (*n*=35).

The escape latency test was done to validate mice competency in spatial learning and memory after honey supplementation.  Figure 3 shows the result for escape latency during the acquisition phase using the spatial hidden platform test. From the graph, during the six days learning phase, results showed a nonsignificant (*p* > 0.05) decrease in latency between control and all treated groups mice from day 1 to day 6. The post hoc test also showed no significant difference (*p* > 0.05) between the control group and all treated groups. Thus, there was no considerable difference found between control group mice and treated group mice during six days of the learning process, which indicated that all mice were competent for spatial learning experiments.

### 3.2. Spatial Hidden Platform Test (Spatial Working Memory)

Following validation, the mice were then tested for spatial working memory on the 7^th^ day through the same spatial hidden platform test. In this memory test, all mice from every group were released from the same starting position for three trials, and their average escape latency to the platform was then compared between the control and each honey-treated group.

**Figure 4:**
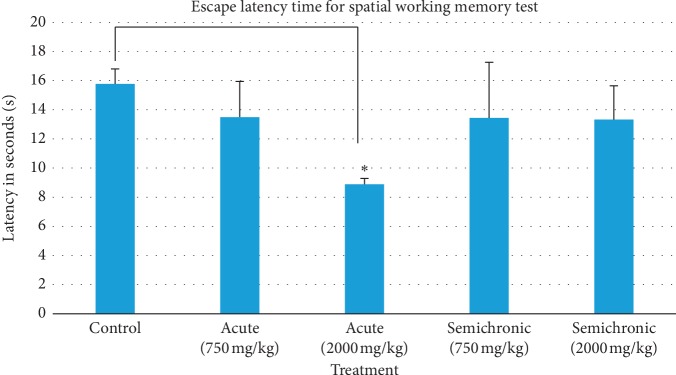
Escape latency for memory test in control and honey-treated groups using the spatial hidden platform test. Data are represented as mean ± SEM, (*n*=35). There was a significant decrease in latency between the control and acute (2000 mg/kg) honey-treated group as *p* < 0.05.

Figure 4 shows the result of escape latency for memory test on the seventh day. In detail, results show that the latency (mean ± SEM) in the control, acute (750 mg/kg), acute (2000 mg/kg), semichronic (750 mg/kg), and semichronic (2000 mg/kg) was 15.78 ± 1.03, 13.50 ± 2.45, 8.89 ± 0.40, 13.44 ± 3.82, and 13.33 ± 2.31 seconds, respectively. Analysis of results by an independent *t*-test between control and each honey-treated group mice provided a significant reduction (*p*=0.000) of escape latency time in the acute (2000 mg/kg) group as compared with control, hence suggesting improvement in spatial working memory. However, further analysis by ANOVA did not reveal a statistically significant difference between groups (*F*(4,30)=1.499, *p*=0.228).

Dose-dependent effects of honey supplementation in all honey-treated group mice were also evaluated in this experiment using the results of escape latency for the memory test, as shown in Figure 4. Further analysis of results between two dosages of acute- and semichronic-treated mice had both shown a nonsignificant decrease (*p* > 0.05) in latency between the higher dosages (2000 mg/kg) of honey-treated mice as compared with the lower dosage (750 mg/kg). Hence, both results showed that there was no considerable difference found for spatial working memory performance in both acute- and semichronic-treated mice when supplemented with different honey dosages. Therefore, there was the possibility that supplementation at a low dosage of 750 mg/kg may already affect the learning and memory performance of honey-treated mice, but additional dosages up to 2000 mg/kg did not offer significant additional effects.

### 3.3. Probe Trial Test (Spatial Reference Memory)

**Figure 5:**
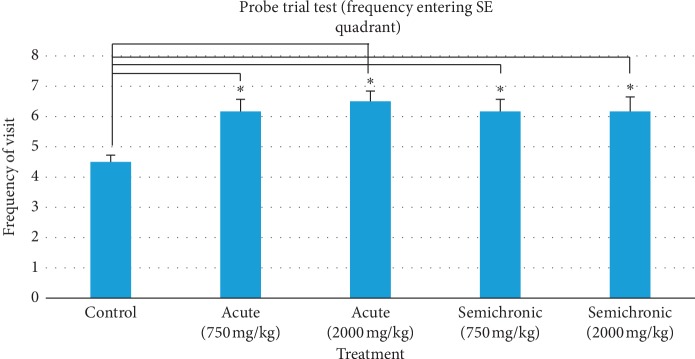
Frequency of visit by control and honey-treated group mice in the target quadrant during the probe trial test. Data are represented as mean ± SEM, (*n*=35). There was a statistically significant difference between the groups as determined by one-way ANOVA (*F*(4,25)=4.419, *p*=0.008). (^*∗*^) indicates significant difference by post hoc test between control group and acute, 750 mg/kg (*p*=0.034), acute, 2000 mg/kg (*p*=0.008), semichronic, 750 mg/kg (*p*=0.034), and Semichronic, 2000 mg/kg (*p*=0.034) group as *p* < 0.05.

The spatial reference memory of honey-treated mice was tested using a probe trial test, which was done on the 8th day of the MWM experiment. The test consists of the elements of frequency visit and time spent in the quadrant where the platform was originally located. Results are summarised in Table 3. The frequency visit (mean ± SEM) in the control, acute (750 mg/kg), acute (2000 mg/kg), semichronic (750 mg/kg), and semichronic (2000 mg/kg) was 4.50 ± 0.22, 6.17 ± 0.40, 6.50 ± 0.34, 6.17 ± 0.40, and 6.17 ± 0.48, respectively. Consistency in the frequency of visit is clearly observed in all honey-treated groups. There was a statistically significant difference between the groups observed (*F*(4,25)=4.419, *p*=0.008). The post hoc test also showed a significant difference between the control group and acute, 750 mg/kg (*p*=0.034), acute, 2000 mg/kg (*p*=0.008), semichronic, 750 mg/kg (*p*=0.034), and semichronic, 2000 mg/kg (*p*=0.034) group as *p* < 0.05 as summarised in Figure 5.

**Figure 6:**
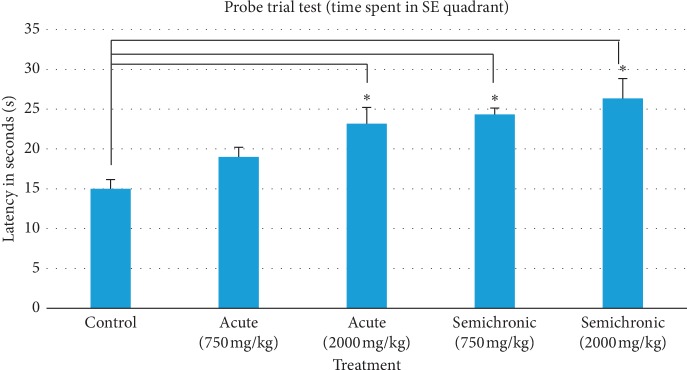
Time spent by control and honey-treated group mice in the target quadrant during the probe trial test. Data are represented as mean ± SEM, (*n*=35). There was a statistically significant difference between groups as determined by one-way ANOVA (*F*(4,25)=7.459, *p*=0.000). (^*∗*^) indicates significant difference by post hoc test between control group and acute, 2000 mg/kg (*p*=0.015), semichronic, 750 mg/kg (*p*=0.004), and semichronic, 2000 mg/kg (*p*=0.001) group as *p* < 0.05.

This frequency result was then further supported with calculated time spent in the target quadrant during the probe trial test as shown in Table 3. The time spent (mean ± SEM) in the control, acute (750 mg/kg), acute (2000 mg/kg), semichronic (750 mg/kg), and semichronic (2000 mg/kg) was 15.00 ± 1.15, 19.00 ± 1.21, 23.17 ± 2.04, 24.33 ± 0.80, and 26.33 ± 2.50 seconds, respectively. There was a statistically significant difference between the groups (*F*(4,25)=7.459, *p*=0.000). The post hoc test also showed significant difference between the control group and acute, 2000 mg/kg (*p*=0.015), semichronic, 750 mg/kg (*p*=0.004), and semichronic, 2000 mg/kg (*p*=0.001) group as *p* < 0.05 as summarised in Figure 6. Notably, prolonged intake of the honey as shown by semichronic supplementation increased spatial reference memory.

### 3.4. Memory-Associated Gene Expression Analysis

**Figure 7:**
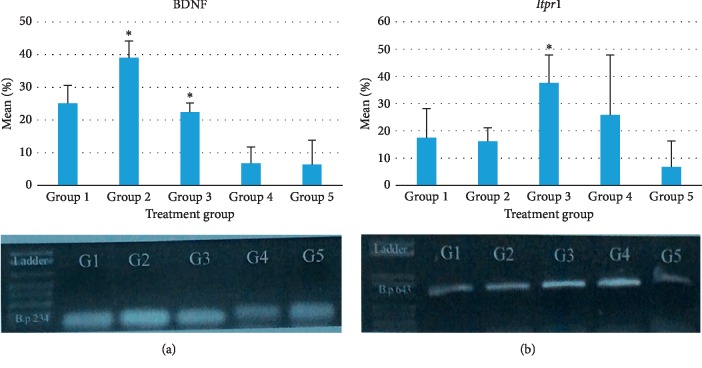
Representative chart of percentage changes in gene expression of BDNF and *Itpr1*. The data were presented as mean (SD) with the respective band of interest normalized by internal control *β-actin*. (^*∗*^) indicates significant differences with *p* < 0.05 when compared with the control group and between treatment groups.

This study evaluated BDNF and *Itpr1* gene expression to investigate the influence of stingless bee honey in the brain of treated mice. Tables 4 and 5 show the results of gene expression levels in the striatum after the stingless bee honey treatment. Results were also showed in a representative image of gel electrophoresis (PCR) bands for each gene expression and summarized in a bar chart as shown in Figure 7.

Overall, results in Tables 4 and 5 show that the gene expression in the mice striatum had significantly changed between the groups for both BDNF and *Itpr1* as *p* < 0.05. Further testing was then conducted to seek the specific groups that show significance using Tukey's post hoc test. The results in Figure 7 show that the gene expression of acute treatment group mice had significantly increased for both BDNF and *Itpr1*. Group 2 (Acute, 750 mg/kg) showed significant upregulation of BDNF as compared with the control and other groups. Group 3 (Acute, 2000 mg/kg) showed significant upregulation of *Itpr1*. However, when comparing the results of honey-treated groups, there was a decrease observed in the BDNF expression of the semichronic-treated mice as compared with the acute-treated mice, whereas the expression of *Itpr1* was more varied within groups.

### 3.5. Metabolomic Analysis

**Figure 8:**
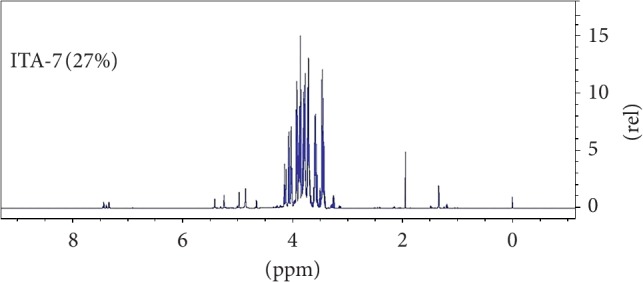
^1^H NMR spectra of the analysed stingless bee honey sample with 27% water content.

Result of water content analysed obtained for the honey sample was 27%.  Figure 8 shows the complete ^1^H NMR spectra of the analysed stingless bee honey sample. Results showed that there was an intensive spectral signal within *δ* 3.3–4.2 ppm spectral regions which were mainly dominated by the major monosaccharide and disaccharide compounds. Other less intensive resonances were also observed in the *δ* 0.0, 1.2–2.0, 4.7–5.4, and 7.3–7.5 ppm regions in the resulting ^1^H NMR spectra of the honey.

**Figure 9:**
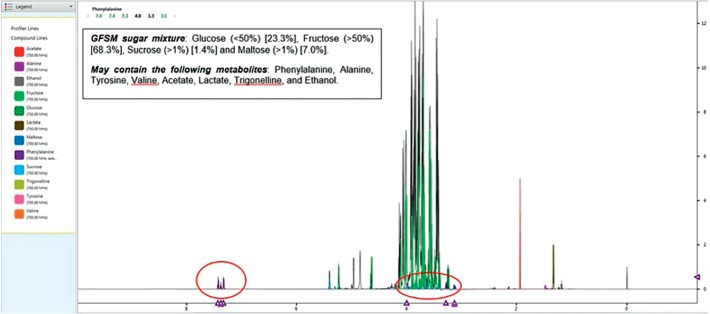
Identification by targeted profiling of 12 compounds of honey from the 700 MHz ^1^H NMR spectra of the analysed stingless bee honey sample. The circled peaks (dark purple signal) represent the ^1^H signal of phenylalanine.

The resulting spectral data were then further analysed with targeted profiling for identification of main constituents found in the honey compound. This was achieved by applying TSP as the internal standard. Results obtained from the resulting spectral data of the analysed honey sample identified 12 compounds.  Figure 9 shows the direct identification of 12 compounds tested from the 700 MHz ^1^H NMR spectra of the analysed honey sample. Results showed that the main constituent compounds found in the analysed stingless bee honey was GFSM sugar mixture comprising glucose (23.3%), fructose (68.3%), sucrose (1.4%), and maltose (7.0%). Results also showed that the honey sample might contain trace amounts of phenylalanine, alanine, tyrosine, valine, acetate, lactate, trigonelline, and ethanol metabolites. Results in Figure 9 also highlighted the presence of phenylalanine in the spectral regions of *δ* 3.0–4.0 ppm and *δ* 7.0–7.5 ppm.

## 4. Discussion

Learning and memory are continuous processes where knowledge is acquired in learning and then encoded, stored, and later retrieved through memory processing. Short-term memory or working memory has a limited capacity and only lasts for a short time, whereas long-term memory can be stored in a more substantial capacity and may have unlimited duration [34]. Spatial learning involves the formation of two subtypes of spatial memory: spatial working memory and spatial reference memory [35]. Spatial memory is a type of episodic memory because it is a conscious collection of the past events. It is also proposed to be structured as detailed or schematic coarse topographic representations of the environment that provide sufficient cues to support navigation [36].

In this study, several behavioural paradigms of MWM were used to validate and select the competitive animals and subsequently to measure the spatial learning and memory of mice after stingless bee honey supplementation. In summary, the spatial hidden platform test was used to evaluate spatial learning and spatial working memory, while the probe trial test was used to evaluate spatial reference memory. This MWM test allows accurate testing throughout the study of spatial learning and memory because animals used a spatial strategy to reach the target platform location using information learned based on the spatial configuration of distal cues [34]. The acquisition trials are proposed to allow spatial learning through allocentric navigation using surrounding distal cues, while the probe trial test is proposed to examine if the trained animal is using distal cues to form a spatial map to locate the hidden platform after various trials [37]. Via the acquisition phase, mice learn the location of the hidden platform based on distal cues around the pool and their escape latency to reach the platform will decrease [34]. Results as in Figure 3 show that all mice from every group tested (honey-treated and control) were capable of spatial learning, and no mice needed to be excluded in the next behavioural testing.

After validation, the mice were then further tested for spatial working memory on the seventh day using the same spatial hidden platform test. This memory test was evaluated for spatial working memory performance as all mice were released from the same starting position for three trials, and the average escape latency between control and honey-treated groups were compared. Results shown in Figure 4 indicate that there is a significant decrease (*p* < 0.05) in the escape latency time for acute (2000 mg/kg) honey-treated group mice as compared with the control, hence suggesting an improvement in the spatial working memory of the mice. However, in a doses dependent manner, results indicate that an increase in honey dosage for both acute- and semichronic-treated mice does not give a significant additional improvement to spatial working memory performance among the honey-treated mice.

On the eighth day of the MWM experiment, the mice were tested for spatial reference memory using the probe trial test. Frequency of visit and time spent in the quadrant of the previous platform (target quadrant, SE) were used as quantitative data for measuring spatial reference memory because the mice were still depending on their memory of surrounding distal cues learned from the previous acquisition testing to escape. Both results as shown in Figures 5 and 6 indicate that all honey-treated mice significantly visited and spent more time in the target quadrant as compared with control group mice (*p* < 0.05), indicating an increase in spatial reference memory performance. Both semichronic-treated mice also had better performance than acute-treated mice in prolonged supplementation. Therefore, these findings suggest that the stingless bee honey supplementation may positively affect and increase the learning and memory performance, especially on this spatial reference memory.

After the behaviour test, further evaluation was done on the mice's brain using histology analysis; results did not reveal any improvement of cell number or neuronal plasticity and yet did not exhibit any signs of neurodegenerative effects to the brain cells. This indicates that high dose honey supplementation up to 2000 mg/kg did not result in any drawback.

**Figure 10:**
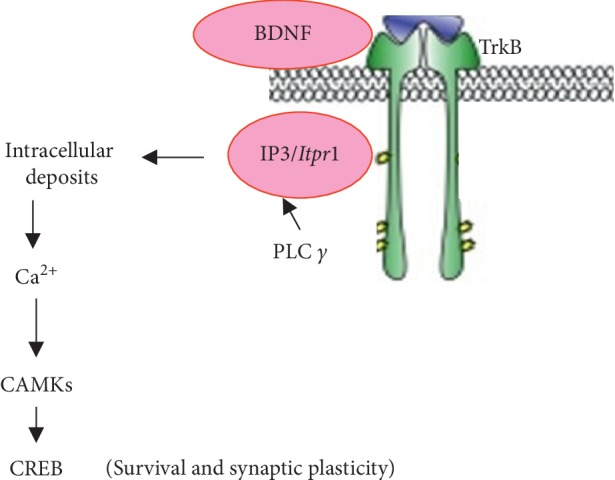
Mechanism of BDNF signalling through TrkB receptor.

In the mechanism of learning and memory processes, neurotrophin signalling through TrkB receptors regulates proliferation, cell survival, dendrite and axon growth, and the fate of neural precursors [17, 18]. The BDNF binds to TrkB receptor and controls plasticity and synaptic transmission and promotes neuronal differentiation and survival. This mechanism of BDNF-TrkB signalling is being studied most extensively in the adult central nervous system (CNS) [38] and plays a role in long-term potentiation (LTP) [19]. As summarised in Figure 10, when BDNF docks with the Trk receptor, PLC *γ* is phosphorylated, which causes a breakdown of lipids to *Itpr1*/IP_3_ and promotes an increase in diacylglycerol (DAG) and intracellular Ca^2+^ concentration [32]. BDNF release is supported by the BDNF-induced increase in the NMDAR subunit, creating more opportunity for Ca^2+^ influx. The IP_3_ activity and intracellular Ca^2+^ release are then boosted when there is a rise in TrkB signalling, thus contributing to activation of the CAMK enzymes and enhancement of the CREB cycle [18]. Concisely, BDNF gene promotes expression by upregulating the phosphorylation of CREB through BDNF-TrkB signalling, thus enhancing memory [39]. The BDNF signalling pathways help to activate camp response element-binding protein (CREB) transcription factors and CREB-binding proteins (CBP) [17] and leads to synaptic plasticity. In contrast, a disruption on BDNF deficits shows a significant impairment of LTP in BDNF-knockout mice but were then rescued upon viral-mediated re-expression of the BDNF gene or upon acute treatment with recombinant BDNF [40].

In this study, results of gene expression analysis as in Figure 7 showed that the BDNF and *Itpr1* gene expressions in mice striatum were affected following honey treatments. The significant upregulation of both genes were detected from the acute-treated group of mice. Hence, the results may indicate that supplementation with stingless bee honey leads to specific upregulations of gene expression tested (BDNF and *Itpr1*), which may lead to the improvement of memory in mice. However, the results also showed a decrease of BDNF expression in semichronic-treated mice. Hence results may indicate that the upregulation of the genes after seven days of supplementation does not last for prolonged supplementation up to 35 days.

In the metabolomics analysis of stingless bee honey, the NMR spectroscopy results identified four amino acids. Amongst these amino acids, phenylalanine drew our attention. Several studies had suggested that phenylalanine may give memory-enhancing effects because it can act directly on BDNF to improve brain signalling and physiology [41, 42]. Observations from a previous study also highlighted the consistency of the phenylalanine in local stingless bee honey [33]. Phenylalanine is a dietary amino acid that is converted to tyrosine which occurs concurrently with the conversion of tetrahydrobiopterin to 4a-hydroxytetrahydrobiopterin and is catalysed by phenylalanine hydroxylase [43]. Tyrosine is needed in the brain as it is the main constituent of tyrosine receptor kinase B (TrkB) which plays a role as BDNF receptor.

Therefore, all findings indicate a possibility that the presence of phenylalanine in the stingless bee honey sample may have contributed to the upregulation of BDNF in the honey-treated mice, which then leads to improvements in their spatial memory performance in the behaviour test. Thus, future study is proposed using a specific BDNF blocker to elucidate the central mechanism of phenylalanine interaction with BDNF.

## 5. Conclusions

The results, especially on spatial reference memory, suggest that stingless bee honey supplementation in Swiss albino mice positively affects to acutely improve memory. High-dose supplementation at 2000 mg/kg which is equivalent to a human dose of 162 mg/kg showed strong capabilities of the stingless bee honey to improve spatial working memory whilst prolonged intake up to 35 days increased the spatial reference memory. This is possibly associated with the presence of phenylalanine in the stingless bee honey, which directly upregulates the BDNF and *Itpr1* genes in the mice brain as observed in gene expression analysis. This study provides the behavioural evidence of the memory-enhancing effects of local-harvested stingless bee honey on mice. Future studies with specific BDNF inhibitors are recommended to further confirm the underlying mechanism of honey in learning and memory.

## Figures and Tables

**Table 1:** Summarization of experimental groups.

Groups	Concentration of stingless bee honey (mg/kg)	Duration of supplementation (days)
1 Control	Ultrapure water	NA
2	750	7
3	2000	7
4	750	35
5	2000	35

**Table 2:** List of specific primers with its sequences.

*BDNF*	
Forward	5′-TAACTAGGAAAACTTGGGACCCTTC-3′
Reverse	5′-TGGTGGAACTTTACGACACCA-3′

*Itpr1*	
Forward	5′-CCAGCCTGTCTTTGTGCAAC-3′
Reverse	5′-TTCAAGCTCCTGCTCTGTGC-3′

**Table 3:** Results summarised for the probe trial test.

Probe trial test	Control	Acute (750 mg/kg)	Acute (2000 mg/kg)	Semichronic (750 mg/kg)	Semichronic (2000 mg/kg)
*Frequency of visit*	4.50 ± 0.22	6.17 ± 0.40	6.50 ± 0.34	6.17 ± 0.40	6.17 ± 0.48
*Time spent in platform-quadrant (s)*	15.00 ± 1.15	19.00 ± 1.21	23.17 ± 2.04	24.33 ± 0.80	26.33 ± 2.50

**Table 4:** BDNF gene expression levels in the striatum after stingless bee honey treatment.

Group	Dosage (mg/kg)	Treatment period (days)	Mean (SD)	*X* ^2^ statistics (df)^*α*^	*p* value
1	—	—	25.09 (5.48)	12.37 (4)	<0.05
2	750	7	39.00 (5.13)		
3	2000	7	22.42 (2.78)		
4	750	35	6.78 (4.97)		
5	2000	35	6.34 (7.53)		

**Table 5:** *Itpr1* gene expression levels in the striatum after stingless bee honey treatment.

Group	Dosage (mg/kg)	Treatment period (days)	Mean (SD)	*X* ^2^ statistics (df)^*α*^	*p* value
1	—	—	17.43 (10.80)	9.87 (4)	<0.05
2	750	7	16.20 (4.91)		
3	2000	7	37.54 (10.35)		
4	750	35	25.84 (22.02)		
5	2000	35	6.78 (9.56)		

## Data Availability

The data used to support the findings of this study are available from the corresponding author upon request.
